# The Effect of a Diet Supplemented with Organic Minerals and l-Carnitine on Egg Production and Chemical Composition and on Some Blood Traits of Pheasant Hens (*Phasianus colchicus*)

**DOI:** 10.3390/ani13213428

**Published:** 2023-11-06

**Authors:** Edyta Kowalczuk-Vasilev, Marian Flis, Agata Bielak, Renata Klebaniuk, Dariusz Gugała, Mirosław Karpiński, Grzegorz Rytlewski, Eugeniusz R. Grela

**Affiliations:** 1 Institute of Animal Nutrition and Bromatology, Faculty of Animal Sciences and Bioeconomy, University of Life Sciences in Lublin, 20-950 Lublin, Poland; edyta.kowalczuk@up.lublin.pl (E.K.-V.); agata.bielak@up.lublin (A.B.); renata.klebaniuk@up.lublin.pl (R.K.); eugeniusz.grela@up.lublin.pl (E.R.G.); 2 Department of Animal Ethology and Wildlife Management, Faculty of Animal Sciences and Bioeconomy, University of Life Sciences in Lublin, 20-950 Lublin, Poland; gugaladariusz@op.pl (D.G.); miroslaw.karpinski@up.lublin.pl (M.K.); 3 Polish Hunting Association, District Board Gdańsk, 80-286 Gdańsk, Poland; goigr@wp.pl

**Keywords:** pheasant hens, chelates, calcitriol, l-carnitine, eggs, blood

## Abstract

**Simple Summary:**

Raising and breeding pheasants is becoming more and more popular, both among hunters and consumers of meat and eggs. The nutritional value of these products, as well as the suitability of eggs for reproduction, is determined by the quantity and quality of dietary nutrients, including minerals, vitamins, and amino acids. In the current study, we have examined the combined effect of a partial replacement of Ca, Fe, Cu, and Zn inorganic salts with their glycine complex and calcitriol addition, with or without the l-carnitine supplementation. We hypothesized that introducing l-carnitine along with glycine chelates and calcitriol may potentiate the effect of these two nutritional interventions on some reproductive traits, the blood lipid and mineral profile, and the mineral and fatty acid profile of pheasant egg yolk. The proposed nutritional supplementation of the pheasant’s diet contributed to an increase in the number of laid eggs, with a simultaneous decrease in the number of rejected eggs, which is crucial for improving pheasant rearing and positively affected the quality of eggs; therefore, it might be a good strategy for increasing the nutritional reserves of poultry and their reproduction.

**Abstract:**

The study aimed to determine the effect of replacing 75% of inorganic calcium, iron, zinc, and copper salts with organic forms (glycine chelates of these elements) with or without the addition of l-carnitine on some reproductive traits and the blood lipid and mineral profile, as well as mineral and fatty acid profile of pheasant egg yolk. The study was performed on three groups of pheasant hens using glycine chelates with calcitriol (group II) or analogical treatment with the addition of l-carnitine at the level of 100 mg/kg of feed (group III) instead of Ca, Fe, Cu, and Zn salts (control). The replacement of inorganic forms with glycinates contributed to an increase in the number of laid eggs with a concomitant lower share of rejected eggs. The supplementation of organic forms of minerals improved mineral absorption and bioavailability in blood serum as well as in the egg yolk of experimental groups. Egg yolk fat was characterized by a higher proportion of polyunsaturated fatty acids and a favorable ratio of PUFA ω-3/ω-6. The proposed nutritional supplementation of the pheasant’s diet might be a good strategy for increasing the nutritional reserves of poultry and improving their reproduction.

## 1. Introduction

Pheasant breeding is becoming increasingly popular, both among hunters (settlement fisheries) and consumers of meat and eggs [1]. The nutritional value of pheasant products and the suitability of eggs for reproduction is determined by the quantity and quality of dietary nutrients, including minerals and feed additives [2]. The mineral requirements of pheasants reared in captive conditions are covered by the use of salts (i.e., sulphates, carbonates, phosphates) or oxides. In recent years, there has been a trend to replace non-organic sources in poultry diets with organic sources of minerals [3,4,5,6,7]. This approach contributes to better bioavailability of minerals, their increased deposition in bird tissues, and modification of egg mineral composition [3,6,8]. However, utilization of organic minerals is largely dependent on the ligand; therefore, amino acids, e.g., glycine and other small molecules with facilitated access to the enterocyte are supposed to be better utilized by animals. After absorption, organic minerals may show physiological effects that improve specific metabolic responses and increase the deposition of minerals in yolk and eggshells. According to Bhoyar [4], utilization of chelated trace minerals (Zn, Cu, and Mn with hydroxyl-methionine) resulted in optimum production of quality eggs, with improved hatchability and chick quality. The bioavailability of some minerals, like calcium and phosphorus, is influenced also by the presence of vitamin D [4,9,10]. To be metabolically active, vitamin D needs to be converted-first into the 25-hydroxycholecalciferol (calcidiol) form in the liver, and subsequently in the kidneys to turn into its active metabolite 1,25-dihydroxycholecalciferol (calcitriol) [11]. The results of our previous studies have shown that direct supplementation of the active form of vitamin D can support mineral metabolism in pheasants [6,12].

The metabolism of nutrients, and thus the direction of the deposition of protein, fat, and other components in the animal organism and the products obtained from them (meat and eggs) can be significantly influenced by some feed additives such as probiotics, synbiotics, eubiotics and others [2]. An interesting additive may be l-carnitine, which has an amino acid structure (it is formed from lysine and methionine) but has a vitamin function [13,14,15]. It participates in lipid metabolism and the transport of branched amino acids, is a donor of the acetyl groups in the synthesis of acetylcholine, and plays an important role in detoxification processes [13,16]. The compound is formed in the liver and kidneys. These organs have all the enzymes needed for its synthesis, but, apart from the mentioned amino acids, iron (Fe^2+^), ascorbic acid, pyridoxine (B6) and niacin (PP) are essential. Their absence or deficiency in feed leads to a reduction in the endogenous synthesis of carnitine. The addition of l-carnitine may modify lipid metabolism and the fatty acid profile in muscles and eggs [13]. l-carnitine plays a crucial role in transporting long-chain fatty acids across the inner mitochondrial membrane [17]. Its function is partly focused on energy production through β-oxidation, and thus l-carnitine supplementation may accelerate fat metabolism in the yolk and expedite follicular development. l-carnitine may also increase metabolic rates in the magnum and shell gland, thereby resulting in albumen deposition and shell calcification, followed by an increase in egg weight [18]. It shows the ability to reduce blood levels of triacylglycerols and cholesterol [16].

Our previous study [6,12] proved a beneficial effect of the partial replacement of some inorganic minerals with the glycine complex (50% and 75% replacement of inorganic salts) and calcitriol addition on performance, slaughter traits, and sensory and physicochemical characteristics of pheasant meat, as well as on the performance, hatching, minerals, and fatty acid composition of eggs. We hypothesized that introducing l-carnitine along with glycine chelates and calcitriol may potentiate the effect of these two nutritional interventions. The changes in the mineral composition of eggs and shells caused by the addition of organic minerals and modification of the metabolism by the addition of l-carnitine may lead to the production of eggs with increased nutritional value and better hatchability, and consequently to higher embryo survival and more efficient rearing of pheasant chicks. Therefore, this study aimed to determine the effect of replacing 75% of calcium, iron, zinc, and copper nonorganic salts with organic forms (glycine chelates of these elements) with or without the addition of l-carnitine on some reproductive traits, the lipid profile, and the mineral content of blood and the minerals and fatty acid profile of pheasant egg yolk. 

## 2. Materials and Methods

### 2.1. Diet and Experimental Design 

Copper, zinc, iron, and calcium formulated as salts and glycine chelates were introduced into the mineral and vitamin premix which did not contain these minerals. The Cu, Zn, Ca, and Fe requirements in the feed mixtures were calculated based on the dietary recommendations for poultry NRC [19]. The Glystar Forte chelates by Arkop Sp. z o.o., containing 16% Zn and 36% glycine, 16% Cu and 37% glycine, 16% Fe and 42% glycine, and 20% Ca and 46% glycine were used in the experiment (Table 1). The l-carnitine in the form of Carniking (Lonza Ltd., Basel, Switzerland) was added in an amount of 100 mg to 1 kg of the mixture. Active vitamin D (calcitriol) in the form of Rovimix Hy-D (DSM Switzerland) was used in the diet for experimental groups (II and III). All the feed components were mixed and pelleted (0.5 mm diameters) at 60 °C.

The study was carried out on 24 16-week-old healthy pheasants (21 females and 3 males) from the breeding flock of the experimental station in their first reproductive season. The birds (one cock and seven hens per cage) were divided into three experimental groups (Table 1). A natural mating system was also used in each cage (feeding group) consisting of one male and seven females. The birds were kept outdoors, in cages of the following size: 8.5 m length × 5.0 m width × 3.5 m height. Each cage was equipped with two nipple drinkers, together with an automatic feeder (40 cm long, i.e., 4.0 cm of feeder edge per bird). The pheasants in each group had constant access to the feed and water (supplied with stoneware drinkers). 

The detailed ingredient and chemical compositions of the experimental basic diets during the rearing period between 5 and 16 weeks of bird life and during laying are presented in the Flis and Gugała [5] publication. The hens’ diet was administered to the breeding pheasants for 4 weeks before the laying period (20 weeks). 

### 2.2. Sampling

Pheasant eggs were collected daily and placed in trays and kept at 18 °C for 7 days. The eggs collected during the week were weighed with an electronic scale (precision balance, Balance XPR404S, Mettler-Toledo LLC, Columbus, OH, USA) with an accuracy of ±0.01 g. In order to check the correct formation of eggs for hatching before and during the incubation process, the eggs are scanned to assess the presence of the embryo and then eliminated if the embryo is missing, defective, or dead. The same procedure is followed for the various incubation periods of the eggs and their destination for hatching. 

Six eggs during five weeks from each weekly egg collection were taken from each group for analytical processing. From the eggs collected (30 pieces from each group), the yolk was separated. The average pooled samples for analysis were obtained from 3 eggs (*n* = 10). Egg yolk was mixed and frozen at −20 °C until laboratory analyses. Blood samples (3 mL) for biochemical and mineral examinations were sampled twice (in the 18th and 20th week of the birds’ life) from the vena basilica of the left wing (*n* = 14 per group) and collected using syringe needle assemblies that had been flushed with heparin. The heparinized blood was immediately centrifuged at 837× *g* at 4 °C for 10 min, and plasma samples were stored at −20 °C in 1.5 mL test tubes until the analyses were performed.

### 2.3. Laboratory Analysis

The chemical compositions (dry matter, crude protein, crude fiber, crude fat, and crude ash) of the basal diets were analyzed according to AOAC procedures [20]. Nutrient content in yolk egg (dry matter, crude protein, and crude ash) was analyzed according to AOAC procedures [20]. The mineral (Ca, Mg, K, Na, Cu, Fe, and Zn) contents in the feed and yolk egg samples were determined using the AAS flame technique in a Unicam 939 (AA Spectrometer Unicam, Shimadzu Corp., Tokyo, Japan) apparatus, after incineration at 550 °C, according to the methods adopted by AOAC. The total P content in the feed and eggs and yolk egg was identified colorimetrically [21] with a Helios Alpha UV–VIS apparatus (Spectronic Unicam, Leeds, UK). 

Blood plasma without signs of hemolysis was analyzed for the content of total protein, glucose, total cholesterol, high-density-lipoprotein cholesterol fraction (HDL-Chol), and triacylglycerols. Low-density lipoprotein cholesterol (LDL-Chol) content was estimated according to the Friedewald et al. [22] equation. The content of selected minerals was estimated in blood plasma as well by colorimetric methods, according to the manufacturer’s protocol, using reagent kits (BioMaxima, Lublin, Poland) and a biochemical analyzer Metrolab 2300 GL (Metrolab SA, Buenos Aires, Argentine). The analysis procedures were verified with the use of multiparametric control plasma (BioCal) as well as control plasma, with a normal level (BioNorm)and a high level (BioPath) of indices (BioMaxima, Lublin, Poland; Hydrex Diagnostics, Warsaw, Poland).

The total fat of the yolks for cholesterol and fatty acid analysis was extracted with a chloroform/methanol mixture according to the Folch et al. [23] method. The percentage of fatty acid methyl esters was estimated using the gas chromatography procedure on a Varian CP-3800 chromatograph (Varian Inc., Palo Alto, CA, USA). The volume of the injected sample was 1 mL. FAMEs were identified by comparison of retention times with known standards, using a 37 Component FAME Mix (Supelco, Bellefonte, PA, USA). The chromatograph operating conditions for fatty acid separation were as follows: the capillary column CP WAX 52CB DF 0.25 mm of 105 m length, gas carrier—helium, flow rate 1.4 mL/min, column temperature 120 °C, gradually increasing by 2 °C/min up to 210 °C, determination time 127 min, feeder temperature 160 °C, detector temperature 160 °C; other gases—hydrogen and oxygen. 

Lipid quality indices, i.e., atherogenicity index (AI) and thrombogenicity index (TI) were calculated according to the Ulbricht and Southgate [17] equations:

AI = [C12:0 + (4 × C14:0) + C16:0]/[n-6 PUFA + n-3 PUFA + MUFA];

TI = [C14:0 + C16:0 + C18:0]/[(0.5 × MUFA) + (0.5 × n-6 PUFA) + (3 × n-3 PUFA) + n-3/n-6 PUFA].

The hypocholesterolemic/Hypercholesterolemic ratio (h/H) was calculated according to the Fernández et al. [18] formula:

h/H = (C18:1 + C18:2 + C18:3 + C20:3 + C20:4 + C20:5 + C22:4 + C22:5 + C22:6)/(C14:0 + C16:0).

Cholesterol was determined by gas chromatography analysis on a Varian CP-3800 chromatograph (Varian Inc., USA), according to the method developed by Botsoglou et al. [24]. Aliquots of 1 µL from the saponified extracts were injected (splitless mode) in a fused silica capillary column (30 m × 0.53 mm i.d.) coated with a 1.0 µm thick SPB-1 film. The column temperature was programmed from 250 to 300 °C at 10 °C/min and held at 300 °C for 15 min. The injection port and flame ionization detector temperatures were set at 300 °C. The hydrogen carrier gas was set at 3.4 mL/min. All the injections were made in the splitless mode. Cholesterol was identified by comparing sample retention times to those of an authenticated laboratory standard. Quantification was carried out against an external standard based on a curve plotted with cholesterol levels and peak area values. Cholesterol concentrations were expressed as mg/g yolk.

### 2.4. Statistical Analysis

The results were subjected to statistical analysis using Statistica 10.0 software, in which mean values in groups and standard error of the mean (SEM) were obtained. Differences between mean values were determined with the multiple range test (analysis of variance ANOVA, α = 95%; *p* < 0.05), and their significance was verified with Tukey’s post hoc test.

## 3. Results

Supplementation of 75% of Ca, Fe, Cu, and Zn salts with glycinates and calcitriol resulted in an increase in the number of laid eggs by 7.9% (*p* = 0.018), with a reduced proportion of rejected eggs. Moreover, the eggs of birds from this group were characterized by lower weight (Table 2). Dietary supplementation with chelates or chelates and l-carnitine (group II vs. III) did not significantly affect egg weight. The combination of chelates with 100 mg of l-carnitine in the pheasant diet (group III vs. I) led to a decrease in NfE in egg yolk (Table 2). The mineral content of egg yolk and serum is shown in Table 3. In the egg yolk of birds of the experimental groups (II and III) an increased content of calcium (*p* = 0.043), iron (*p* = 0.035), and zinc (*p* = 0.023) was found as compared to the control group (Table 3). The combined supplementation of chelates and l-carnitine (group III vs. II) did not cause significant differences in the mineral content of pheasant egg yolk.

Replacement of inorganic forms of Ca, Fe, Cu, and Zn with glycinates in diets for group II pheasants did not cause significant changes in the content of macroelements (Ca, K, P, Na, and Mg) in blood serum (Table 3). However, a significant increase in Fe, Zn, and Cu levels was found in the serum of birds of this group. The addition of l-carnitine (group III vs. II) did not significantly affect the content of analyzed minerals in pheasant blood serum (Table 3).

The use of minerals in the form of glycine chelates (group II vs. I) contributed to an increase in the share of the HDL cholesterol fraction in total cholesterol content, with slight differences in the content of other analyzed indices in laying pheasant blood serum (Table 4). However, the combined addition of chelates and l-carnitine (group III vs. I) resulted in a significant increase in protein levels (*p* = 0.043), a decrease in total cholesterol and LDL fractions, and an increase in HDL fractions. The analysis of pheasant blood serum indices between groups II and III indicated a decrease in total cholesterol and LDL fraction and an increase in HDL cholesterol fraction (Table 4).

No significant changes were found in the fatty acid profile in the egg yolk of birds fed on a diet with mineral chelates, except for an increased proportion of DHA and a favorable PUFA ω-3/ω-6 ratio (Table 5). The addition of l-carnitine to the glycinate diet contributed to a decrease in SFA and an increase in PUFA ω-3 and PUFA ω-6, and a narrowing of the ω-6/ω-3 ratio and the AI, TI, and h/H indices beneficial for human health (Table 5). Eggs of group III pheasants had a reduced cholesterol count in the yolk, but the difference between other experimental groups was not statistically confirmed (*p* = 0.063).

## 4. Discussion

Pheasants are an important bird species in terms of hunting interest. Their meat and eggs are a valuable dietary component in human nutrition [25,26]. Consumers are interested not only in nutrients but also in minerals, as well as the profile of fatty acids, especially the ratio of PUFA and cholesterol [27,28]. Therefore, attempts are being made to use varied feeding conditions, including feed additives that may affect the quantity and quality of eggs and their suitability for roasting, as well as their chemical composition and nutritional value [2,3,5]. Nutritional factors, e.g., supplementation of fat, proteins, minerals, and l-carnitine can modify egg quality by inducing metabolic changes, resulting in a modification of their content of nutrients, including the composition of fatty acids, cholesterol, and minerals [29]. Supplementing the diet with highly bioavailable chelated trace minerals (Zn, Fe, and Cu) allows the bird to obtain the most use of the mineral, compared to supplementing inorganic trace mineral sources.

In poultry production, l-carnitine has a multi-functional purpose, which includes growth promotion, strengthening the immune system, antioxidant effects, and improving semen quality [13,16]. Some studies have shown that supplemental l-carnitine improved body weight gain and reduced the abdominal fat content of broilers [30]. Reports on the effects of dietary l-carnitine on egg production and egg quality of pheasant hens are limited. According to Rabie et al. [31], dietary l-carnitine did not influence laying performance (egg production rate, mean egg weight, daily feed intake, daily egg mass, and feed conversion) or external egg quality as measured by egg weight, egg-shape index or eggshell quality, either measured directly as shell breaking strength or indirectly as shell weight, shell thickness or shell weight per unit surface area.

The weight of eggs of pheasant eggs in the control group was similar to the values obtained in the studies of Kożuszek et al. [32] and Nowaczewski et al. [33] and the mineral content in the publication by Genchev [28]. The replacement of calcium, copper, zinc, and iron salts with glycine chelates of these elements in the amount of 75% introduced along with a calcitriol addition contributed to an increase in the amount of obtained eggs (354 vs. 328) and a decrease in the number of rejected eggs, but with reduced egg weight. But on the whole, it seemed the addition of l-carnitine in Group III had the strongest effect on the studied traits.

The addition of l-carnitine to the diet with mineral chelates did not significantly affect the weight and morphological characteristics of pheasant eggs, but improved the hatching and survival of chicks up to 14 days of age. This is crucial for the aviary rearing of pheasants, due to the still unsatisfactory results of their reproduction. The low reproduction indices often seen in pheasant breeding might be associated with the stress the birds are exposed to due to changing, sometimes unfavorable, atmospheric conditions and to the observed growing intensification of rearing [33]. In both experimental groups, there was about a 10% increase in the number of eggs laid, with a ca. 50% reduction in rejected eggs as compared to the control group. Simultaneously, in both groups an increase in UFA ω-3 was stated. The observed increased number of laid eggs might be attributed to the role of omega-3 fatty acids in reproductive activities [34,35]. This impact might be affected by the addition of l-carnitine. It plays a pivotal role in transporting long-chain fatty acids across the inner mitochondrial membrane [17]. Due to its role in energy production through B-oxidation, l-carnitine supplementation may therefore accelerate fat metabolism in the yolk, and thus expedite follicular development [17,35].

In the present study, no significant changes in the nutrient content of the whole egg and yolk itself were found, and only a decrease in NfE content was noted. Partial salt supplementation with calcium, copper, zinc, and iron chelates only revealed a tendency to reduce cholesterol levels in egg yolk, but with a significant increase in the HDL fraction of blood serum. In the available literature, there are different values of cholesterol levels in the blood serum and eggs of pheasants. The cholesterol level in eggs changes with species, breed, the hen’s age, egg and yolk weight, and diet [36]. The cholesterol content according to Aygün and Olgun [27] was 159.7 mg/dl, Kaźmierska et al. [26] gave 6.81 mg/g yolk, while Choi et al. [25] gave 18.8 mg/g yolk and Mangiagalli et al. [1] 5.3 mg/g whole egg. Nowaczewski et al. [33] determined cholesterol to be at the level of 15.2–17.2 mg/g yolk, depending on the maintenance method (cages vs. aviary) or the color of the shell. Parizadian et al. [37] reported a reduction in cholesterol from 32.5 mg/g to 22.5 mg/g egg yolk with the addition of 125 mg of l-carnitine to 1 kg feed for Japanese quail laying hens. Supplementing the diet with l-carnitine will reduce blood triglyceride and cholesterol and improve egg quality in laying Japanese quail. These results suggest that l-carnitine may have hypocholesterolemic effects, and differences in blood cholesterol levels may be due to changes in thyroid activity, which plays an important role in the metabolism and seems to be stimulated by l-carnitine [16,37]. l-carnitine has probably induced a reduction in the hepatic biosynthesis of yolk cholesterol, or an alteration in the transport from the liver to the ovarian follicle and the oocyte. l-carnitine plays a crucial role in fatty acid metabolism by directing fatty acids into the mitochondrial oxidative pathway through the action of specialized acyltransferases [38]. 

An increased content of iron and zinc in the egg yolk of group II was observed. Also, in the blood serum, an increased content of iron, zinc, and copper was found. The observed increased amounts of these elements were caused by their better availability from the mineral chelates used in the feed [4,7]. Nowaczewski et al. [33] found the most potassium and calcium in whole eggs (without shell), followed by sodium and magnesium, not giving the value of phosphorus, while for microelements, the most was for iron and zinc. The content of these elements depended on the color of the shell and the system of keeping the pheasant laying hens. The content of minerals in egg yolks and blood serum was not determined by the addition of l-carnitine. The addition of l-carnitine to the diet, with calcium, copper, zinc, and iron glycinates, reduced the content of total cholesterol, especially the LDL fraction, and increased the HDL cholesterol fraction, which is a desirable phenomenon in human consumption of pheasant eggs. It should also be mentioned that the obtained pheasant blood serum index is similar to the values given by Nazifi et al. [39] and Schumann et al. [40].

The most oleic (18:1, ω-9), palmitic (16:0), and linoleic (18:2, ω-6) acid is present in the egg yolk of farmed pheasants, which is confirmed by the studies of Choi et al. [25], Mangiagalli et al. [1], and Nowaczewski et al. [33]. There are also polyunsaturated acids of the ω-3 family (ALA, EPA, and DHA), but their proportion is below 2% of the sum of fatty acids. The dietary supplementation for pheasants with calcium, copper, zinc, and iron glycinates has not contributed to significant changes in the fatty acid profile of pheasant eggs. However, the addition of l-carnitine in group III of the pheasants had a significant impact on the FA profile of the egg yolk. In this group, the highest share of polyunsaturated fatty acids was observed. The proportion of C 22:6 (DHA) acid was the highest (0.67 vs. 0.48%), and the ratio of ω-3/ω-6 acids in the egg yolk of this group of birds was improved, as compared to the control group. The level of DHA in egg yolk can result from its direct deposition from the diet or a de novo synthesis from its α-linolenic and eicosapentaenoic precursors [41]. The increase in fatty acids can be explained by the preventive role of l-carnitine in the egg yolk against oxidation of the polyunsaturated fatty acids in the egg [35], as the presence of l-carnitine in the diet caused an accumulation of l-carnitine in the egg yolk [16].

Pheasant eggs, with a relatively low fat content and cholesterol percentage as compared with the eggs of other bird species, are considered a valuable product from a human nutrition point of view [25,42]. It is worth emphasizing that pheasant eggs are richer than chicken eggs in essential amino acids and some minerals. The inclusion of organic forms of minerals in the current study increased the content of Ca, Fe, and Zn in the egg yolk of both experimental groups, compared to the control group. Furthermore, the addition of l-carnitine has contributed to the reduction in SFA, especially palmitic acid, which has a hypercholesterolemic potential, and the increase in polyunsaturated fatty acids, especially PUFA ω-3, which improved the ω-3/ω-6 ratio and improved the values of h/H, AI and TI indices of pheasant egg yolk. The lower the values of the AI and TI indices, the more beneficial they are for human health, as they are correlated with the increased risk of cardiovascular diseases.

## 5. Conclusions

The replacement of inorganic forms of minerals with their chelated forms, supported by the concomitant addition of calcitriol and l-carnitine, resulted in improved lipid and mineral metabolism. In the egg yolk of pheasants, a higher content of crude ash and calcium, zinc, iron, and copper was found. Egg yolk fat was characterized by a higher proportion of DHA acid and a favorable ratio of PUFA ω-3/ω-6. In the fatty acid profile of egg yolk, a reduced proportion of SFA, in particular palmitic acid, an increased ratio of PUFA ω-3 and ω-6, and a beneficial-for-human-health index h/H and TI and AI were found.

The proposed nutritional supplementation of the pheasants’ diet contributed also to an increase in the number of laid eggs, with a simultaneous decrease in the number of rejected eggs, which is crucial for improving pheasant rearing. 

Therefore, the 75% replacement of inorganic salts with glycine chelates and the addition of an active form of vitamin D in the amount of 2500 IU, along with 100 mg/kg of feed of l-carnitine, might be a good strategy to increase the nutritional reserves of poultry and their reproduction. 

## Figures and Tables

**Table 1:** The composition of mineral and l-carnitine addition in 1 kg of experimental diets.

Item	Requirement NRC, 1994	Treatments		
		I—Control	II—Chelates	III—Chelates + l-Carnitine
Calcium carbonate (35% Ca), g	25	25 (100%) ^(1)^	-	-
Calcium glycine ^(2)^ (20% Ca), g		-	18.75 (75%)	18.75 (75%)
Copper sulphate pentahydrate (25% Cu), mg	5	5 (100%)	-	-
Copper glycine (16% Cu), mg		-	3.75 (75%)	3.75 (75%)
Ferrous sulphate heptahydrate (20% Fe), mg	60	60 (100%)	-	-
Ferrous glycine (16% Fe), mg		-	45 (75%)	45 (75%)
Zinc sulphate heptahydrate (22% Zn), mg	50	50 (100%)	-	-
Zinc glycine (16% Zn), mg		-	37.5 (75%)	37.5 (75%)
l-carnitine, mg		-	-	100
Vitamin D_3_, IU kg^−1^	2500	2500	-	-
Calcitriol, IU kg^−1^		-	2500	2500

^(1)^—percent provided of nutritional requirement for the minerals [19]. ^(2)^—Glycinate chelate—Glystar Forte Ca, Cu, Fe, and Zn (Arkop, PL).

**Table 2:** Total number and weight of egg and nutrient contents of egg yolk.

Item	Treatment			SEM	*p* Values
	I—Control	II—Chelates	III—Chelates + l-Carnitine		
Total number of eggs	328 ^b^	354 ^a^	362 ^a^	32.5	0.018
Rejected eggs, %	6.71 ^a^	3.95 ^b^	3.59 ^b^	0.28	0.041
Egg weight, g	33.7 ^a^	32.5 ^b^	32.9 ^b^	1.05	0.043
Chemical composition of the yolk					
Dry matter	47.52	47.45	47.22	0.38	0.189
Crude protein	16.14	16.23	16.36	0.19	0.138
Crude fat	26.35	26.29	26.01	0.16	0.195
Crude ash	1.57	1.65	1.69	0.07	0.063
Nitrogen-free extract	3.46 ^a^	3.28 ^ab^	3.16 ^b^	0.12	0.036

^a, b^—values with different letters differ significantly at *p* ≤ 0.05.

**Table 3:** Mineral content in yolk eggs and blood of pheasant hens.

Minerals	Tissue	Treatment			SEM	*p*-Value
		I—Control	II—Chelates	III—Chelates + l-Carnitine		
Calcium	Blood, mmol/L	3.38	3.47	3.46	0.12	0.193
	Yolk, mg/kg	1620.4 ^b^	1743.5 ^a^	1733.4 ^a^	35.8	0.043
Phosphorus	Blood, mmol/L	2.35	2.32	2.34	0.09	0.217
	Yolk, mg/kg	5214.5	5223.1	5232.5	84.2	0.226
Magnesium	Blood, mmol/L	1.32	1.35	1.36	0.06	0.222
	Yolk, mg/kg	155.2	154.6	155.4	2.16	0.441
Potassium	Blood, mmol/L	3.78	3.81	3.79	0.08	0.337
	Yolk, mg/kg	1182.6	1201.3	1194.5	39.2	0.295
Sodium	Blood, mmol/L	146.8	145.4	149.4	5,95	0.124
	Yolk, mg/kg	576.2	569.4	567.9	21.3	0.289
Iron	Blood, mmol/L	28.82 ^a^	31.48 ^b^	31.54 ^b^	1.02	0.029
	Yolk, mg/kg	62.5 ^b^	71.4 ^a^	72.1 ^a^	5.52	0.035
Zinc	Blood, mmol/L	14.73 ^a^	16.39 ^b^	16.67 ^b^	0.68	0.021
	Yolk, mg/kg	27.8 ^b^	34.5 ^a^	35.1 ^a^	3.84	0.023
Copper	Blood, mmol/L	38.15 ^a^	46.33 ^b^	47.11 ^b^	1.46	0.014
	Yolk, mg/kg	0.92	1.05	1.03	0.05	0.056

^a, b^—values with different letters differ significantly at *p* ≤ 0.05.

**Table 4:** Content of protein, glucose, and lipid profiles (mmol/l) in pheasant hens’ blood.

Item	Treatment			SEM	*p* Values
	**I—Control**	**II—Chelates**	**III—Chelates + l-Carnitine**		
Protein	38.32 ^a^	39.12 ^ab^	40.35 ^b^	0.79	0.043
Glucose	21.34	21.28	21.35	0.38	0.324
Total cholesterol	3.64 ^a^	3.57 ^a^	3.27 ^b^	0.17	0.032
LDL	1.88 ^a^	1.79 ^a^	1.27 ^b^	0.19	0.014
HDL	1.38	1.55	1.58	0.21	0.054
Triacylglycerols	0.84	1.00	0.92	0.17	0.068
% HDL	37.84 ^a^	41.04 ^b^	48.29 ^c^	1.68	0.021

^a, b, c^—values with different letters differ significantly at *p* ≤ 0.05.

**Table 5:** Profile of fatty acid (% of total FA), lipid quality indices, and cholesterol content in pheasant egg yolk.

Fatty Acids	Treatment			SEM	*p* Values
	I—Control	II—Chelates	III—Chelates + l-Carnitine		
C 12:0	0.01	0.01	0.01	0.001	0.678
C 14:0	0.52	0.51	0.48	0.05	0.062
C 16:0	26.37 ^a^	26.17 ^a^	24.91 ^b^	0.89	0.031
C 17:0	0.18	0.17	0.19	0.01	0.226
C 18:0	8.92	8.83	8.64	0.38	0.094
C 20:0	0.02	0.03	0.05	0.02	0.055
**∑ SFA**	36.02 ^a^	35.72 ^a^	34.28 ^b^	1.12	0.031
C 16:1, ω-7	0.41	0.42	0.43	0.05	0.107
C 16:1, ω-9	6.11	6.17	6.21	0.29	0.179
C 17:1, ω-7	0.12	0.13	0.12	0.02	0.106
C 18:1, ω-7	2.78	2.81	2.72	0.21	0.144
C 18:1, ω-9	37.32	37.25	37.47	1.02	0.137
C 20:1, ω-9	0.28	0.29	0.27	0.02	0.093
**∑ MUFA**	47.11	47.15	47.31	1.22	0.107
C 18:2	13.85 ^a^	13.92 ^a^	14.97 ^b^	0.73	0.028
C 20:2	0.17	0.18	0.18	0.01	0.202
C 20:4	0.82	0.81	0.85	0.11	0.114
**∑ PUFA** ω-6	14.84 ^a^	14.91 ^a^	16.00 ^b^	0.84	0.017
C 18:3	1.02 ^a^	1.09 ^ab^	1.16 ^b^	0.05	0.041
C 20:5 (EPA)	0.11	0.12	0.14	0.02	0.098
C 22:5	0.09	0.11	0.12	0.02	0.082
C 22:6 (DHA)	0.48 ^a^	0.56 ^b^	0.67 ^c^	0.06	0.024
**∑ PUFA** ω-**3**	1.70 ^a^	1.88 ^ab^	2.09 ^c^	0.19	0.036
ω-3/ω-6	0.115 ^a^	0.126 ^b^	0.131 ^c^	0.02	0.027
ω-6/ω-3	8.73 ^a^	7.93 ^b^	7.66 ^c^	0.19	0.021
h/H	2.10 ^a^	2.12 ^ab^	2.29 ^b^	0.06	0.034
AI	0.45 ^a^	0.44 ^a^	0.41 ^b^	0.02	0.045
TI	0.99 ^a^	0.97 ^ab^	0.89 ^b^	0.04	0.036
Cholesterol, mg/g of yolk	10.32	10.21	9.85	0.42	0.063

^a, b, c^—values with different letters differ significantly at *p* ≤ 0.05.

## Data Availability

Data are contained within the article.
